# Supplementary material for: Patterns of Intron Gain and Loss in Fungi
Source: PLoS Biol. 2004 Nov 30;2(12):e422. doi: 10.1371/journal.pbio.0020422 (PMC532390; doi:10.1371/journal.pbio.0020422)
Supplement: Table S1 — Also available at http://genes.mit.edu/NielsenEtAl/. (4.3 MB ZIP). [file pbio.0020422.st001.zip › NielsenEtAl/html/1409.html]

AN8013.1.NCU01174.1.MG08648.1.FG06781.1


```
 CLUSTAL W (1.82) Multiple Sequence Alignments - Introns Inserted


Sequence 1: MG08648.1	697 aa
Sequence 2: FG06781.1	638 aa
Sequence 3: NCU01174.1	728 aa
Sequence 4: AN8013.1	672 aa
Alignment Length: 758 aa
Number Identitical Residues: 255 aa
Alignment Score (without introns) 13000


MG08648.1 	--MIT-----AQLREALQEAVVMCSERCLYQSAK----------W2AAELLSAIPP---D
NCU01174.1	-MLTLFSSFDPSASSNLQHHNNNSQKRAFANGNGQGLPRFSICFM2AAELLDSVPDPVLS
FG06781.1 	--MVLSAHEISQLRAALQDAVVKCSERCLYQSSK----------W2AAELLNALPE---T
AN8013.1  	MASNFSEDNIKELKYRLEAAAVQCSERCLYQSAK----------W2AAEILDSIIPIDNY
          	           .    *:     ..:*.: :.              ***:*.::      

MG08648.1 	TNDAE-PEGDQSRFISPGFARNPDPAEAALEAAEFNKYLLAKSFFDCKEFDRCAAVFLPD
NCU01174.1	DLDQKEAVAGLQGYQHPAVSANPDPVEAELEAKELSKYLLAKSFFDCKEFDRCASVFLPD
FG06781.1 	DDDEENINPADPSHVSPIFAANNDPEEAILEARELSKYLLAKSLFDCREYDRCAAVFLPD
AN8013.1  	DTDPDSPMDITEPRPQNPYLRTQDPVEAALEAQEYHKYLLAKSYFDTREYDRCASVFLPP
          	  * ..               . ** ** *** *  ******* ** :*:****:**** 

MG08648.1 	SLLAGLVPMTRAEDNIPS----ANASKDQAKPPPTQTLRPQDLPELSQKSLFLALYAKVI
NCU01174.1	SLLSGLLSTKATDATTPK----GKAKASSAP--PYEQALP----SLSQKSLFLALYAKVI
FG06781.1 	SLLS-SVLASRVDHASAS----SAAGKGKSK--ASEAAVP--LPKLSQKSLFLALYAKFM
AN8013.1  	TISPVSLSTSSPQSKLKSSLRNRKSQGSPHAGLKDNDLKRSPYPKLSQKSLFLALYAKYL
          	:: .  :  .  :    .:  .  :  .       :    .  ..************* :

MG08648.1 	SGEKRKDEDAEMVMGPHDLGTIVNKQLLPVSNYLEQWFNRRSNEDGYTPGSQGWLEYL2Y
NCU01174.1	SGEKKKNEESEMIMGPQDLGTIKNSHLVVVSRFLERWFAERKAEDSDLPPSQGFLEYL2Y
FG06781.1 	SGEKRRNEDSEMVMGPQDLGTVTNKQLLVIGHFLATWFEERTTEDDEVLGSQGWLEYL2Y
AN8013.1  	AGEKRKNEETEMVLGPADGGATVNRELPDLARGLEGWFAERQ-EKGVEDQNHGWLEYL~Y
          	:***:::*::**::** * *:  * .*  :.. *  ** .*  *..    .:*:**** *

MG08648.1 	GMVLAKSKSEAEAMEWFIRSVHLFPMNWGCWLEMTSLISRVAD0LNAISPQLPQNIVSFM
NCU01174.1	GMVMVKEKNEDAALDYLLNSVHLFPWNWGAWLELTNLISGVEQ~LNKITPVLPQNIMSFI
FG06781.1 	GMVLAKEKNDDKALEYFIRSVHKYTMNWGCWLEMTSLISRVED0----------------
AN8013.1  	GVILIKGRNEEEARKWLIRSVHLNPFHWGAWQELNDLLASTED0LKQVVEHLPQNIMTLI
          	*::: * :.:  * .:::.***  . :**.* *:..*:: . :  .      ...  :  

MG08648.1 	FHLHTSLELYQQGPQLAHSLEELLKLFPTSSFLLTCRALLEYHNKD~LTLAEQHFSTLLA
NCU01174.1	FYANTAVNLYQQGQDLAASVNDLLKLFPTSSFILTCRALLNYHSKD~LYTAEQNFSNLLA
FG06781.1 	------------GPGLANSLEQLLSIFPTSSFLLTCNALLAYHAKD~LMAAEQHFTRLLA
AN8013.1  	FHVHCSQELYQATEDTYQTLSELENIFPTSAFLKTQRALLYYHSKD1FEEASHIFTDILI
          	     : .  .       ::.:* .:****:*: * .*** ** ** :  *.: *: :* 

MG08648.1 	LHPHRLDSLDHYSNILYVLNYRPKLAFLAHLCSSVDKFRPESCVVVGNYYSLLSMHEKAV
NCU01174.1	LHPHRLDSLDHYSNILYVLNMRPKLAFLAHLCSSIDKFRPESCVVIGNYYSLLSLHEKAV
FG06781.1 	LHPHRLDSLDHYSNILYVLNLRPKLAFLAHLCSSVDKFRPESCVVIGNYYSLLSMHEKAV
AN8013.1  	TSPHRLDSLDHYSNILYVMGARPQLAFVAQVATATDKFRPETCCVVGNYYSLKSEHEKAV
          	  ****************:. **:***:*::.:: ******:* *:****** * *****

MG08648.1 	QYFRRALTLDRSCLSAWTLMGHEYVELKNTHAAIESYRRAVDVNRRDYRAWYGLGQTYEV
NCU01174.1	QYFRRALTLDRSCLSAWTLMGHEYVELKNTHAAIESYRRAVDVNRRDYRAWYGLGQTYEV
FG06781.1 	QYFRRALTLDRSCLSAWTLMGHEYVELKNTHAAIESYRRAVDVNRRDYRAWYGLGQTYEM
AN8013.1  	MYFRRALTLDRNFLSAWTLMGHEYIEMKNTHAAIESYRRAVDVNRKDYRAWYGLGQAYEV
          	 **********. ***********:*:******************:**********:**:

MG08648.1 	LEMHTYALWYYKKAAGLRPWDGKMWMAVGSCLQKMGRERDGIKALKRALLADSSYETGSG
NCU01174.1	LEMNSYALYYYKKAAGLRPWDGKMWQAVGSCLQKMGKDRDGIKALKRALLADSYYDS-TL
FG06781.1 	LEMHTYSLWYYKKAAGLRPWDGKMWMAVGSCLQKMGRERDGIKALKRALLADAYYDVGSS
AN8013.1  	LDMSFYALFYYQRAAALRPYDPKMWQAVGSCYAKMGRIEQSIKALKRALVAGSYYAEDPS
          	*:*  *:*:**::**.***:* *** *****  ***: .:.********:*.: *  .. 

MG08648.1 	SGLGTAGASGSFTAGGLDRTGALHMDPEVLLQIANMYDNLGELDEARMYMELCVSQED--
NCU01174.1	SSFGSAGPADRMAQ----------MDPEVLLQIASMYDRLGEVEEARAYMELCVAQED--
FG06781.1 	FGSGDLLGSRNATG---------HMDPDILLQIAVMYDQLGEEEEARSYMELCVAQEDGS
AN8013.1  	QHGGRK-----------------ILDPETLYQIATLYERLEDEEEAAAYMELTLQQET--
          	   *                    :**: * *** :*:.* : :**  **** : **   

MG08648.1 	GGEED-------DMAGLGNSISIHRDASVGGSDDDEPRPDGSKMNTTPIVAGGGGTGVTP
NCU01174.1	GGATDNADASAVCTSNPGESFVVHGDSPGSDGDAEGGYEGRGGAGQ------GEGTGVTV
FG06781.1 	GGAAG-------AEVDPGESIAIHNDSPAGSENGAENDENSNNE----------GTGVTA
AN8013.1  	GGQPD-------EVSDVSDSEIEDDQSNTASTSGANQRRARRSPNDDE-EEAYHGTGPTA
          	**  .          . .:*   . ::  .. .           .      .  *** * 

MG08648.1 	ATSKAR~MWLA----------RHALEYEDYAEAHRLATELCQDGVEVEEAKALIREARAQ
NCU01174.1	ATSKAP1MIRIGSNGGGGPKLRRVPRSQRYYENISTIPPLTDQGSGIYEDPSQESDDDDD
FG06781.1 	ATSKAR~MWLA----------KFSMRTGDYMTASRLAGELCQDGVEVEEAKALVREVRSR
AN8013.1  	TTSKAR~LWLA----------RWSLRHGDLERADQLAGELCQDGVEVEEAKALMRDVRAR
          	:****  :             :   .             * ::*  : *  :   :    

MG08648.1 	MDLAQAQGDGAGSGGALSRTPQQARG-----------------
NCU01174.1	DDDENGGNDRHDDWSGEDYLTDGYDDDDVDDDDDDNGGAPTNA
FG06781.1 	MEATGMLDP-------LS-------------------------
AN8013.1  	REAGA--------------------------------------
          	 :
```
